# Fucoidan Elevates MicroRNA-29b to Regulate DNMT3B-MTSS1 Axis and Inhibit EMT in Human Hepatocellular Carcinoma Cells

**DOI:** 10.3390/md13106099

**Published:** 2015-09-24

**Authors:** Ming-De Yan, Chih-Jung Yao, Jyh-Ming Chow, Chia-Lun Chang, Pai-An Hwang, Shuang-En Chuang, Jacqueline Whang-Peng, Gi-Ming Lai

**Affiliations:** 1 Cancer Center, Wan Fang Hospital, Taipei Medical University, Taipei 11696, Taiwan; E-Mails: yanmd717@gmail.com (M.-D.Y.); yao0928@tmu.edu.tw (C.-J.Y.); jqwpeng@nhri.org.tw (J.W.-P.); 2 Department of Internal Medicine, School of Medicine, College of Medicine, Taipei Medical University, Taipei 11031, Taiwan; E-Mail: chow0803@yahoo.com.tw; 3 Comprehensive Cancer Center, Taipei Medical University, Taipei 11031, Taiwan; 4 Division of Hematology and Medical Oncology, Department of Internal Medicine, Wan Fang Hospital, Taipei Medical University, Taipei 11696, Taiwan; E-Mail: 101255@w.tmu.edu.tw; 5 Seafood Technology Division, Fisheries Research Institute, Council of Agriculture, Keelung 20246, Taiwan; E-Mail: ampere0808@msn.com; 6 National Institute of Cancer Research, National Health Research Institutes, Miaoli County 35053, Taiwan; E-Mail: sechuang@nhri.org.tw

**Keywords:** fucoidan, hepatocellular carcinoma, miR-29b, DNMT3B, MTSS1, EMT

## Abstract

Accumulating evidence has revealed that fucoidan exhibits anti-tumor activities by arresting cell cycle and inducing apoptosis in many types of cancer cells including hepatocellular carcinoma (HCC). Exploring its effect on microRNA expression, we found that fucoidan markedly upregulated miR-29b of human HCC cells. The induction of miR-29b was accompanied with suppression of its downstream target DNMT3B in a dose-dependent manner. The reduction of luciferase activity of DNMT3B 3′-UTR reporter by fucoidan was as markedly as that by miR-29b mimic, indicating that fucoidan induced miR-29b to suppress DNMT3B. Accordingly, the mRNA and protein levels of MTSS1 (metastasis suppressor 1), a target silenced by DNMT3B, were increased after fucoidan treatment. Furthermore, fucoidan also down-regulated TGF-β receptor and Smad signaling of HCC cells. All these effects leaded to the inhibition of EMT (increased E-cadherin and decreased N-cadherin) and prevention of extracellular matrix degradation (increased TIMP-1 and decreased MMP2, 9), by which the invasion activity of HCC cells was diminished. Our results demonstrate the profound effect of fucoidan not only on the regulation of miR-29b-DNMT3B-MTSS1 axis but also on the inhibition of TGF-β signaling in HCC cells, suggesting the potential of using fucoidan as integrative therapeutics against invasion and metastasis of HCC.

## 1. Introduction

Hepatocellular carcinoma (HCC), presented with high morbidity and mortality, is the third leading cause of cancer-related deaths worldwide [1,2,3]. In Taiwan, HCC is ranked second leading cause of cancer death due to its high recurrence after resection and poor prognosis [4], which is primarily related to tumor invasion and distant metastasis [5]. Sorafenib, a tyrosine kinase inhibitor (TKI), is the only available agent to treat advanced HCC so far; however, the outcome remains poor due to the toxicity and salvage signaling pathway developing after TKI treatment [6,7]. Alternative therapeutics is urgently needed.

Various signaling and molecular mechanisms have been reported to regulate the metastatic processes of HCC [8]. Recently, microRNAs (miRs) have been demonstrated to play a crucial role in tumorigenesis and metastasis, suggesting that targeting miRNAs could be a promising approach for the treatment of human cancers [9,10]. MiRs are a class of endogenous, small, non-coding regulatory RNAs of approximately 20–25 nucleotides that negatively regulate gene expression by inhibiting translation or inducing mRNA degradation through base pairing with the 3′ untranslated region (3′-UTR) of target messenger RNAs (mRNAs) [11,12,13].

The miR-29 family consists of miR-29a, miR-29b and miR-29c with shared regulatory capacity. Many reports demonstrate that miR-29 family exerts various effects in preventing cancer progression and carcinogenesis, such as apoptosis induction [14], cell cycle regulation, epigenetic modification, and metastasis inhibition [15]. Decreased expression of miR-29 has been reported in multiple cancers, including HCC [16,17]. One important downstream target of miR-29b is the DNMT3B (DNA methyltransferase 3B), which silences tumor suppressors [18]. Many studies have demonstrated the aberrant levels of DNMTs and dysregulation of the DNA methylation status in HCCs [19,20,21,22]. Compared with the noncancerous liver tissue, the expression of DNMT3B in HCCs was significantly higher [23]. In line with this, MTSS1 (metastasis suppressor 1), a novel target of DNMT3B, is repressed in HCC [24,25]. Moreover, Fan *et al.* [25] reported that DNMT3B overexpression was detected in 81.25% of clinical HCC specimens and was negatively associated with MTSS1 in HCC cells and clinical samples. Agents able to modulate the miR-29b-DNMT3B-MTSS1 axis may improve the treatment of HCC.

The potential of natural dietary components as cancer therapeutics has been demonstrated in many studies. Among them, dietary components such as curcumin, resveratrol, genistein, epigallocatechin-3-gallate, indole-3-carbinol, and 3,3′-diindolylmethane have been shown to exert their antiproliferative and/or proapoptotic effects in cancer cells through the regulation of one or more miRs [26]. Natural products represent a fruitful source for searching miR regulators.

Fucoidan is a natural sulfated polysaccharide found in the cell wall matrix of brown seaweed. Structurally, fucoidan is a heparin-like molecule with a substantial percentage of l-fucose, sulfated ester groups, as well as small proportions of d-xylose, d-galactose, d-mannose, and glucuronic acid [27]. Various biological activities of fucoidan, such as antioxidant, anti-inflammatory, antiproliferative, and proapoptotic activities have been reported [28,29]. Recently, the effects of fucoidan against breast and lung cancers have been demonstrated in animal models by Hsu *et al.* [30,31]. In those two studies, degradation of TGF-β receptor (TGF-βR) and thereby inhibition of EMT (Epithelial to Mesenchymal Transition) were observed in fucoidan-treated cancer cells. Zhu *et al.* [32] have shown that fucoidan could inhibit the growth of HCC xenograft in animal, but the underlying molecular mechanisms still remains to be elucidated. In addition to the molecular events described above, it is tempting to investigate the potential of fucoidan in the regulation of miRs to combat HCC and thus further delineate the molecular mechanisms underlying the anticancer effects of fucoidan.

In this study, we explored the effects of fucoidan on the regulation of miR-29b in human HCC cells. We found that fucoidan increased miR-29b to suppress the DNMT3B, which resulted in the upregulation of MTSS1. Moreover, in agreement with that reported by Hsu *et al.* [30,31], fucoidan also down-regulated the TGF-β signaling of these HCC cells. These effects leaded to inhibition of EMT (increased E-cadherin and decreased N-cadherin) and prevention of extracellular matrix degradation (increased TIMP-1 and decreased MMP2, 9), by which the invasion activity of HCC cells was diminished. Our results demonstrate the profound effect of fucoidan not only on the regulation of miR-29b-DNMT3B-MTSS1 axis but also on the inhibition of TGF-β signaling in HCC cells.

## 2. Results

### 2.1. Effects of Fucoidan on the Growth and Clonogenicity of Human HCC Cells

**Figure 1:**
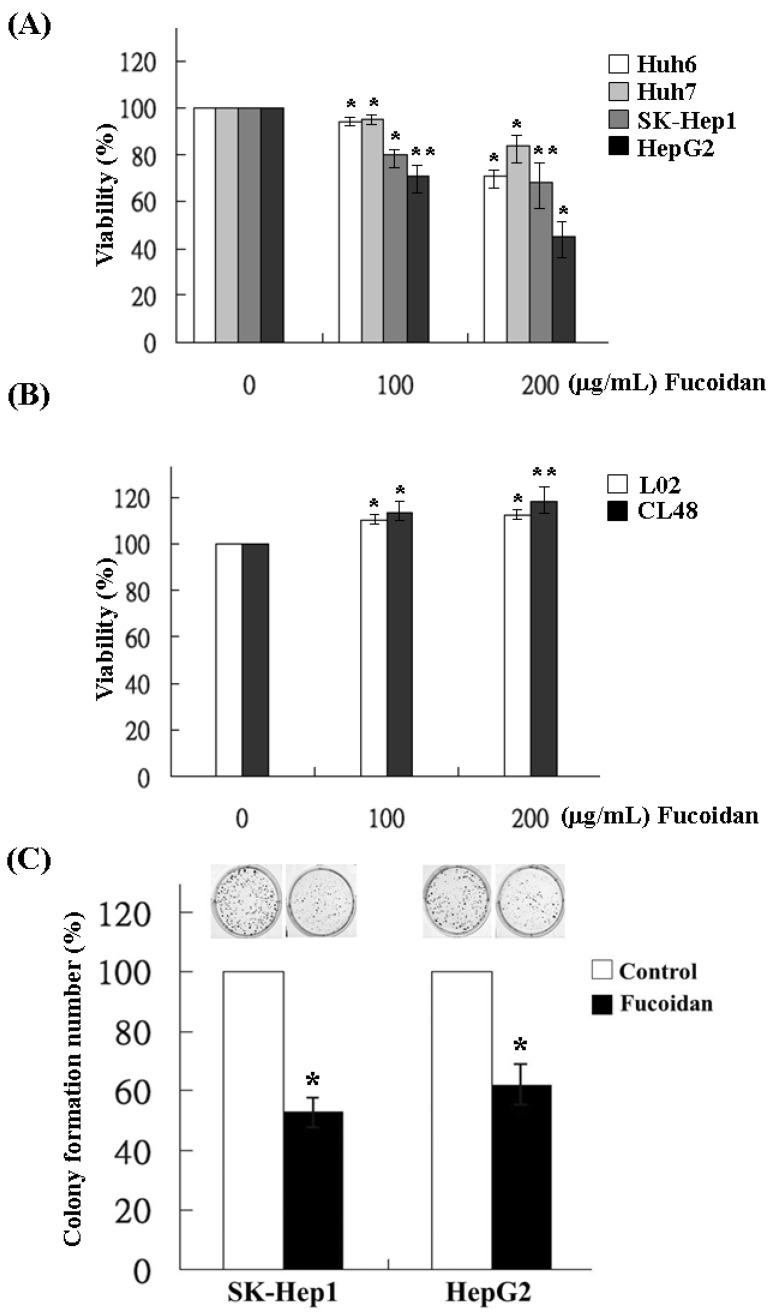
Inhibitory effects of fucoidan on the cell viability and clonogenicity of human HCC cells. (**A**) Four HCC cells and (**B**) two immortalized normal hepatocytes were incubated for 48 h with various concentrations of fucoidan (0, 100 and 200 μg/mL), and the proliferation of cell was measured using MTS (3-(4,5-dimethylthiazol-2-yl)-5-(3-carboxymethoxyphenyl)-2-(4-sulfophenyl)-2H-tetrazolium) assay. Values are expressed as the mean ± standard error of three independent experiments. (**C**) SK-Hep1 and HepG2 cells were treated with fucoidan at 200 μg/mL and the clonogenicity was measured. Each group of fucoidan-treated samples was normalized against each untreated control. The data are representative of three separate experiments and are presented as the mean ± SD; error bars indicate SD. Significant differences are shown (* *p* < 0.05 and ** *p* < 0.01, compared with the control group).

The effects of fucoidan on the growth of HCC cells were determined by MTS assay in Huh6, Huh7, SK-Hep1 and HepG2 cells. Varying degrees of growth inhibition were observed in HCC cells after 48 h treatment of fucoidan. At a dose of 200 μg/mL, fucoidan inhibited 28%, 14%, 31% and 52% of cell growth in Huh6, Huh7, SK-Hep1 and HepG2 cells, respectively (Figure 1A). In contrast, fucoidan had no inhibitory effect on the growth of normal human liver cell lines such as L02 and CL48 at the same dose, suggesting the preferential suppression of cancer cells by fucoidan (Figure 1B). We then examined its effects on the clonogenicity of SK-Hep1 and HepG2 cells, which were relatively more sensitive to fucoidan than the other two HCC cell lines. The result showed that, at a dose of 200 μg/mL, fucoidan significantly decreased the colony formation of SK-Hep1 and HepG2 cells to 55% and 62%, respectively (Figure 1C).

### 2.2. Inhibitory Effect of Fucoidan on Cell Invasion

**Figure 2:**
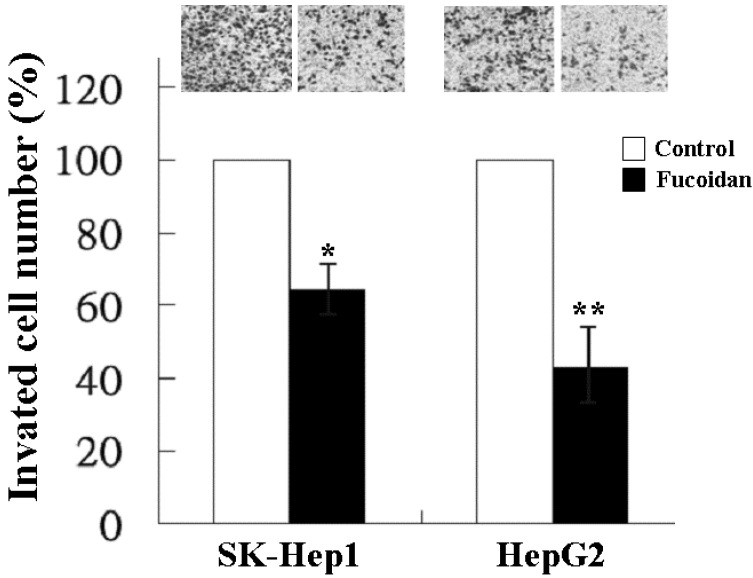
Inhibitory effect of fucoidan on the invasion of human HCC cells. SK-Hep1 and HepG2 cells were treated with fucoidan at a dose of 200 μg/mL for 24 h and the invasiveness was measured by two-chamber transwell assay. Each group of fucoidan-treated samples was normalized against each untreated control. The data are representative of three separate experiments and are presented as the mean ± SD; error bars indicate SD. Significant differences are shown (* *p* < 0.05 and ** *p* < 0.01, compared with the control group).

In addition to tumor growth, invasion and distant metastasis play a crucial role in the poor clinical outcome of HCC. We thus further investigated the effect of fucoidan on the invasiveness of SK-Hep1 and HepG2 cells by the transwell invasion assay. As shown in Figure 2, at a dose of 200 μg/mL, fucoidan significantly reduced the invasion of SK-Hep1 and HepG2 cells to 64% and 44%, respectively.

### 2.3. Fucoidan Increases the Expression of MiR-29b

As regulation of miR has become a novel approach for cancer treatment, we analyzed the miR expression profile of fucoidan-treated HepG2 cells by Affymetrix GeneChip miRNA 2.0 Array. The results showed that fucoidan increased the expression of tumor suppressive miRs such as miR-29 family and miR-1224 (Table 1). The expressions of miR-29b, miR-29a and miR-29c in fucoidan-treated HepG2 cells were increased 8.5-, 7.7- and 7.3-fold, respectively, as compared to the control (Table 1). On the other hand, the expression of oncogenic miRs such as miR-17/92 cluster was markedly suppressed by fucoidan (Table 1).

**Figure 3:**
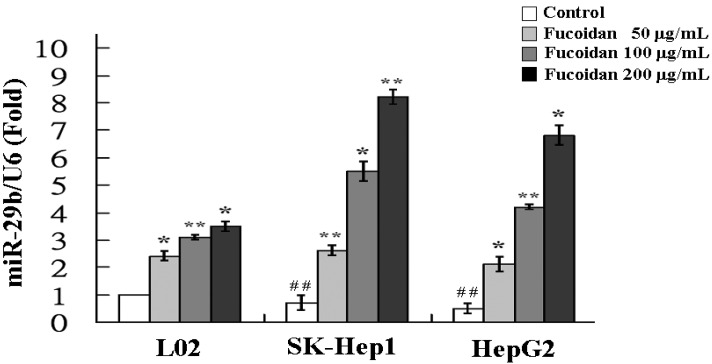
Fucoidan increases the expression of miR-29b. L02, SK-Hep1 and HepG2 cells were treated with fucoidan (0, 100 and 200 μg/mL) for 48 h and the relative miR-29b expression levels were measured by quantitative RT-PCR. Data were normalized to the U6 signal. Each group of fucoidan-treated samples was normalized against each untreated control. The data are representative of three separate experiments and are presented as the mean ± SD; error bars indicate SD. Significant differences are shown (* *p* < 0.05 and ** *p* < 0.01, compared with respective control group; ^##^ *p* < 0.01, compared with the basal level of L02).

The increase of miR-29b expression by fucoidan was further confirmed by real-time quantitative PCR. As shown in Figure 3, the miR-29b expressions in fucoidan-treated normal cell line (L02) and HCC cells were all significantly increased in a dose-dependent manner. At a dose of 200 μg/mL, fucoidan induced 3.5-, 8.2- and 6.8-fold increase of miR-29b expression in L02, SK-Hep1 and HepG2 cells, respectively. The results of HCC cells are compatible to those of miRNA 2.0 array shown in Table 1. Compared to L02, the SK-Hep1 and HepG2 cells possessed significantly lower miR-29b basal levels and the miR-29b induction by fucoidan appeared to be more profound in these two HCC cell lines (Figure 3). It has been shown that miR-29b expression suppressed EMT, angiogenesis, migration and invasion in various types of cancers including HCC [19,33,34]. Considering the marked activation of miR-29 expression by fucoidan in human HCC cells, we investigated the changes of miR-29b downstream target in fucoidan-treated HCC cells to further delineate the underlying molecular events.

**Table 1:** Effects of fucoidan on the microRNA profiling of human HCC cells. Total RNA was isolated from HepG2 cells treated with vehicle or fucoidan at a dose of 200 μg/mL for 48 h. The samples were then analyzed by using Affymetrix GeneChip miRNA 2.0 array (Affymetrix, Santa Clara, CA, USA) containing 4560 probe sets for human small RNAs. The values shown are the increased or decreased fold-changes in expression.

miR-ID	Increased Fold-Changes	miR-ID	Decreased Fold-Changes
miR-29b	8.5	miR-17	15.7
miR-29a	7.7	miR-92a	13.6
miR-29c	7.3	miR-18a	12.0
miR-1224	6.6	miR-192	7.2
miR-133b	2.0	miR-127	2.4
miR-200c	1.7	miR-154	1.9
miR-200a	1.4	miR-21	1.7
miR-205	1.3	miR-680	1.3
miR-208a	1.2	miR-377	1.2
miR-669b	1.2	miR-153	1.1

### 2.4. Fucoidan Increases MiR-29b Expression to Suppress the DNMT3B

**Figure 4:**
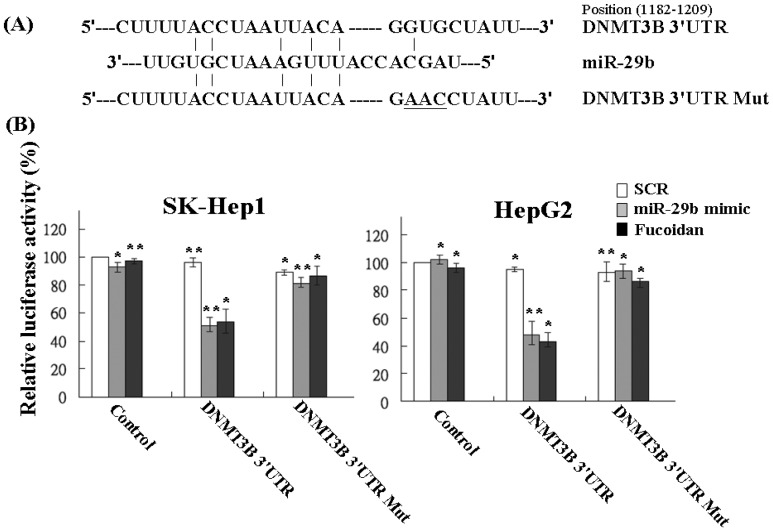
Fucoidan increases miR-29b to target the 3′-UTR region of DNMT3B and repress its expression in human HCC cells. (**A**) Schematic representation of 3′-UTRs of DNMT3B showing putative miR-29b target site; (**B**) After transfection, the DNMT3B-dependent luciferase activity of HCC cells was measured by Dual-Luciferase^®^ Reporter Assay System (Promega, Madison, WI, USA) 48 h after the treatment as indicated. The experiments were performed in triplicate. The values were normalized to the Renilla luciferase activity (transfection efficiency control) and plotted as relative luciferase activity. SCR: scrambled control. Each group of fucoidan-treated samples was normalized against each untreated control. The data are representative of three separate experiments and are presented as the mean ± SD; error bars indicate SD. Significant differences are shown (* *p* < 0.05 and ** *p* < 0.01, compared with the control group).

Using the Pic Tar and TargetScan databases (PicTar: http://pictar.mdc-berlin.de/ and TargetScan: http://targetscan.org/) to predict the targets of miR-29b, the result showed that DNMT3B had the highest score and appeared to be a potential miR-29b target gene. The 3′-UTR of DNMT3B gene matches the seed sequence of miR-29b, representing the putative binding site. To validate the repression of DNMT3B by miR-29b in HCC cells, we created luciferase reporter constructs containing the 300-bp 3′-UTR of DNMT3B (nt 1182–1209 of DNMT3B mRNA) with wild-type or mutated miR-29b-binding site as reported previously (Figure 4A). In agreement with the marked increase of miR-29b expression by fucoidan illustrated in Table 1 and Figure 3, the luciferase activity of the wild-type, but not the mutated, DNMT3B 3′-UTR reporter was obviously repressed to around 50% in SK-Hep1 and HepG2 cells after treatment with fucoidan for 48 h (Figure 4B). As expected, similar and compatible results were observed in these two kinds of HCC cells transfected with miR-29b mimic (Figure 4B), indicating that fucoidan increased miR-29b expression to negatively regulate DNMT3B in HCC cells.

### 2.5. Fucoidan Increases Expression of Metastasis Suppressor 1 (MTSS1), a Novel Target of DNMT3B, Accompanied with EMT Inhibition

**Figure 5:**
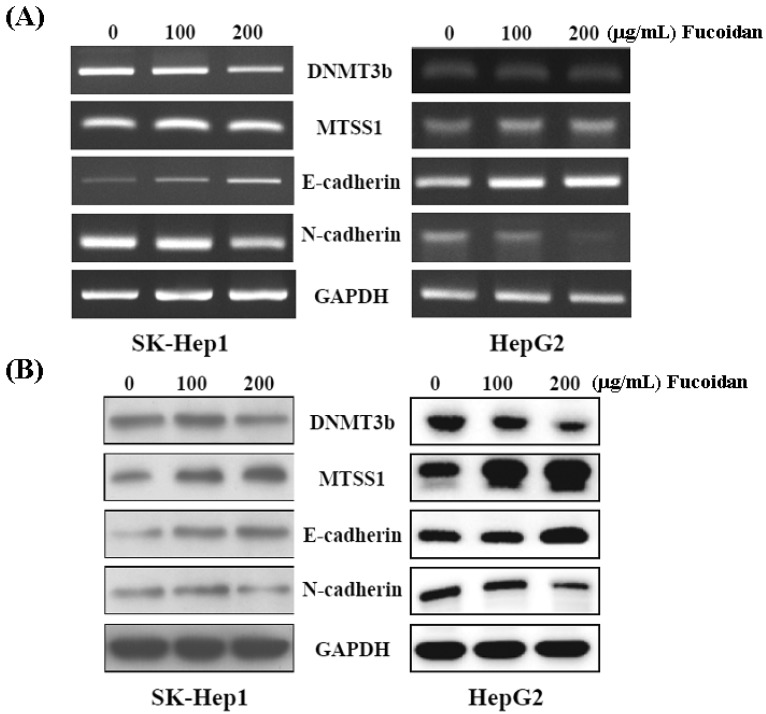
Fucoidan regulates the DNMT3B-MTSS1 axis to inhibit EMT in HCC cells. After treatment with fucoidan for 48 h, the mRNA (**A**) and protein (**B**) levels of DNMT3B, MTSS1, E-cadherin, and N-cadherin were analyzed by RT-PCR and Western blot, respectively. GAPDH (glyceraldehyde-3-phosphate dehydrogenase) was used a loading control.

Western blot and RT-PCR were performed to confirm the inhibitory effect of fucoidan on DNMT3B shown in Figure 4B. As shown in Figure 5, both mRNA and protein levels of DNMT3B were dose-dependently repressed in fucoidan-treated SK-Hep1 and HepG2 cells. Consistently, fucoidan dose-dependently increased the mRNA and protein levels of MTSS1, while repressing the DNMT3B in these HCC cells. As Dawson *et al.* [35] reported that loss of MTSS1 in cancers may diminish the junction stability, which ultimately promotes EMT and metastasis, we then examined the EMT markers in these HCC cells with increased MTSS1. In line with the study by Dawson *et al.* [35], the increase of E-cadherin and reciprocal decrease of N-cadherin represent the inhibition of EMT in these fucoidan-treated HCC cells.

### 2.6. Fucoidan Suppresses TGF-β Signaling Pathway of HCC Cells and Prevents Extracellular Matrix Degradation

**Figure 6:**
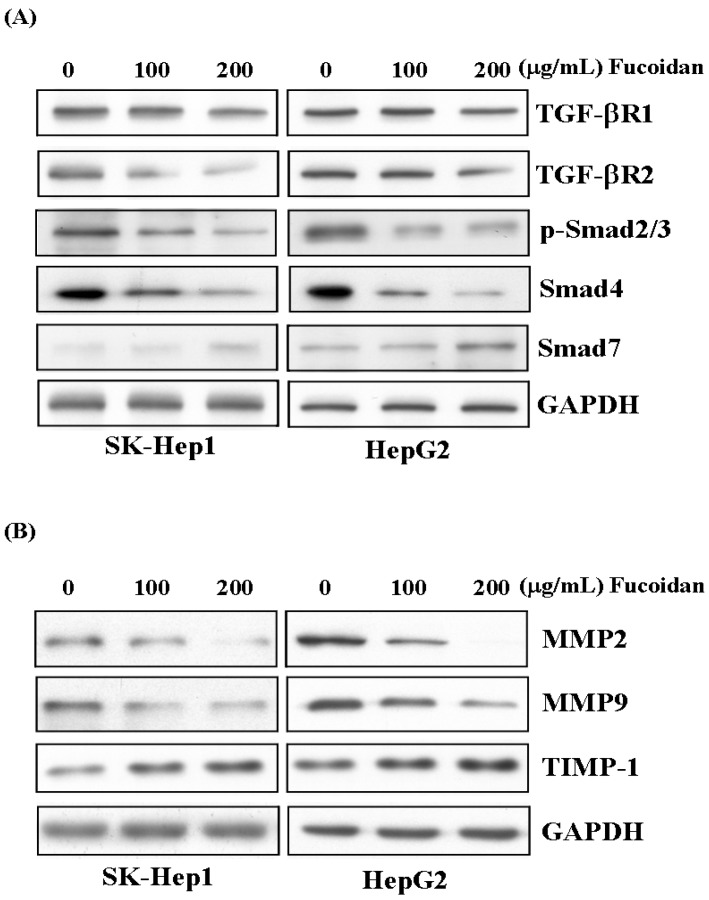
Fucoidan suppresses TGF-β signaling pathway of HCC cells and prevents degradation of extracellular matrix. (**A**) After 48 h of treatment, fucoidan dose-dependently down-regulated the TGF-β receptor 1, 2 (TGF-βR1, 2), phospho-Smad2/3 (p-Smad2/3) and Smad4 protein levels but increased the inhibitory Smad protein, Smad7. (**B**) Fucoidan dose-dependently decreased the protein levels of MMP2 and MMP9 while increased that of tissue inhibitor of metalloproteinase-1 (TIMP-1). The HCC cells were treated with fucoidan (0, 100, 200 μg/mL) for 48 h, and the cell lysates were then collected for Western blotting. GAPDH (glyceraldehyde-3-phosphate dehydrogenase) was used as a loading control.

Like its effects in breast and lung cancer cells, as reported by Hsu *et al.* [30,31], fucoidan also suppressed the TGF-β signaling of these HCC cells. After 48 h of treatment, fucoidan dose-dependently down-regulated the TGF-β receptor 1, 2 (TGF-βR1, 2), phospho-Smad2/3 (p-Smad2/3) and Smad4 protein levels but increased the inhibitory Smad protein, Smad7 (Figure 6A). We then further investigated its impact on the MMPs and TIMP. As shown in Figure 6B, fucoidan dose-dependently decreased the protein levels of MMP2 and MMP9, while increased that of tissue inhibitor of metalloproteinase-1 (TIMP-1). These effects would also contribute to the diminished invasive activity of HCC cells shown in Figure 2.

**Scheme 1:**
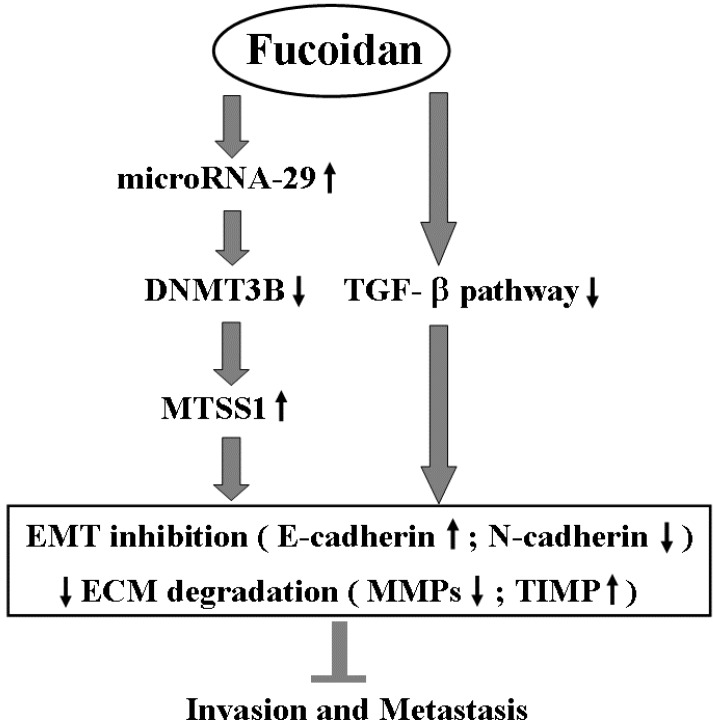
The proposed molecular mechanism of action related to the inhibition of invasion and metastasis of HCC cells by fucoidan. EMT: epithelial to mesenchymal transition; ECM: extracellular matrix.

Besides inhibiting TGF-β signaling, fucoidan profoundly regulates the miR-29b-DNMT3B-MTSS1 axis. These effects would lead to inhibition of EMT (increased E-cadherin and decreased N-cadherin) and prevention of extracellular matrix degradation (increased TIMP-1 and decreased MMP2, 9), and then suppress the migration and invasion of HCC cells. This proposed molecular mechanism of action of fucoidan is illustrated in Scheme 1.

## 3. Discussion

Fucoidan, the major component of brown seaweed, is a multifunctional molecule with anti-tumor potential [28]. The development of fucoidan as a marine anticancer agent has been carried on in many preclinical studies in recent decades. Various anticancer mechanisms such as induction of cell cycle arrest, apoptosis, anti-angiogenesis and immune system activation have been proposed [29]. The effects against different types of HCC cells by fucoidan have been shown *in vivo* and *in vitro* [32]; nevertheless, the underlying molecular events are largely unexplored. This is the first study demonstrating the regulation of miR expression by fucoidan to activate tumor suppressive cascade in HCC cells.

MiR-29b is the most highly expressed miR-29 family member (miR-29a/b/c) and its aberrant expression is common in the majority of human cancers [36]. In HCC, reduced expression of miR-29b was found in clinical samples and significantly associated with worse disease-free survival of patients [14]. By directly targeting the matrix metalloproteinase-2 (MMP-2) expression, miR-29b has been shown to suppress angiogenesis, invasion, and metastasis of HCC in animal model and confirmed in clinical samples [33]. Overexpression of miR-29b has also been reported to suppress MMP-2 level in prostate and lung cancer cells [37,38]. In accordance with these studies, we find that fucoidan induces miR-29b and suppresses MMP-2 in HCC cells.

Furthermore, our results demonstrate that miR-29b directly represses DNMT3B expression by binding to its 3′-UTR region, indicating the repression of DNMT3B by fucoidan in HCC cells is through miR-29b induction. In support of this, miR-29b was recently proved to be an epi-miR that targets epigenetic enzymes such as DNA methyltransferases (DNMTs), leading to the re-expression of tumor suppressors [36]. Induction of miR-29b by curcumin to decrease DNMT3B and epigenetically regulate PTEN in hepatic stellate cells was also reported, representing a novel mechanism for the suppression of liver fibrosis [39]. MTSS1 (metastasis suppressor 1), a downstream target of DNMT3B, was found to function as a tumor suppressor in HCC by Fan *et al.* [25] In line with this finding, our study shows the repression of DNMT3B accompanied with elevation of MTSS1 by fucoidan inhibits growth and invasion activities of HCC cells.

MiR-29b is generally recognized as an important regulator of EMT, a pathway involved in cancer invasion and metastasis [36]. Impact on DNMT3s through miRs-29 has recently been shown to regulate EMT in hepatocytes [19]. Ectopically expressing miR-29b enhances epithelial marker E-cadherin and reduces mesenchymal marker N-cadherin in prostate cancer cells [40]. In agreement with these studies, enhanced E-cadherin and reciprocal reduced N-cadherin were observed in fucoidan-treated HCC cells, suggesting its potential role in miR-29b-mediated EMT inhibition.

Previous studies by Hsu *et al.* [30,31] demonstrated that fucoidan inhibited EMT and invasion of lung and breast cancer cells through degradation of transforming growth factor beta receptor (TGF-βR). In agreement, we found that fucoidan also down-regulated TGF-βR and inhibited the Smad signaling pathway of HCC cells. In addition to Myc and NF-κB, miR-29b expression is also suppressed by TGF-β [36]. As such, it is possible that the induction of miR-29b by fucoidan in HCC cells might attribute to the down-regulation of TGF-βR. Further investigation is worth to delineate the association of fucoidan-induced change of TGF-β signaling and miR-29b expression in cancer cells.

It has been shown that the *in vitro* anticancer activity was significantly higher for low-molecular-weight fucoidan (490 kDa) than for native fucoidan of 5100 kDa [41]. The low molecular weight (around 0.7 kDa) of the fucoidan [42] used in this study might endow the relatively higher anticancer activity that suppressed HCC cells without inhibition on the normal human liver cell lines.

On the other hand, besides the induction of tumor-suppressive miR-29 family, fucoidan also represses the oncogenic miR-17/92 cluster [43], representing its multiple functions in miRs regulation. The miR-17/92 cluster repression might participate in fucoidan-inhibited growth and invasion of HCC cells. Additional investigation is needed. Regarding the important roles of dysregulated miR-29 family and miR-17/92 cluster reported in many cancers [36,43], nutraceuticals able to regulate these miRs will be attractive for integration into conventional cancer therapy. Our results shed light on the application of fucoidan in targeting miRs, which are deregulated in HCC, lung and prostate cancers. Further clinical trials to explore its complementary role as cancer therapeutics are needed.

## 4. Experimental Section

### 4.1. Reagents

The fucoidan powder from *Sargassum hemiphyllum*, a commercial product named Hi-Q Oligo-fucoidan^®^, was provided by Hi-Q Marine Biotech International Ltd. (New Taipei City, Taiwan). It was dissolved in double-distilled H_2_O and stirred at 25 °C for 30 min. The dissolved solution was filtered using 0.22 ìm sterile filters (Millipore, Billerica, MA, USA).

### 4.2. Cell Culture

The human hepatocellular carcinoma cell lines (Huh6, Huh7, SK-Hep1and HepG2) were purchased from the American Type Culture Collection (ATCC) (Manassas, VA, USA). The normal human liver cell lines (L02 and CL48) were obtained from Dr. Ya-Wen Lin’s laboratory (National Defense Medical Center, Taipei, Taiwan). The cell lines were maintained in Dulbecco’s Modified Eagle’s Medium (Gibco, Grand Island, NY, USA) supplemented with 10% fetal bovine serum and 1% Non-Essential Amino Acids (Corning, Cambridge, MA, USA). All cell lines were grown at 37 °C in a humidified 5% CO_2_ atmosphere. These cells were free of mycoplasma contamination.

### 4.3. Cell Viability by MTS Assay

Cell viability was measured by using Cell Titer 96 AQ One Solution (Promega, Madison, WI, USA). Briefly, 3-(4,5-dimethylthiazol-2-yl)-5-(3-carboxymethoxyphenyl)-2-(4-sulfophenyl)-2H-tetrazolium (MTS) reagent (20 μL/well) was added to the cells, followed by incubation for 2 h at 37 °C under humidified 5% CO_2_ in air. After incubation, the absorbance was measured at a wavelength of 490 nm by a PowerWave X Microplate ELISA Reader (Bio-TeK Instruments, Winooski, VT, USA).

### 4.4. Colony Formation Assay

A total of 4 × 10^3^ cells were seeded in six-well tissue culture dishes. After 2 weeks of culture, colonies were fixed with 4% paraformaldehyde and stained by 0.05% crystal violet. The total number of colonies in each well was counted.

### 4.5. Invasion Assay

The invasiveness of the HCC cells was evaluated by a transwell invasion assay. Briefly, the cells were plated in 200 μL serum free medium with or without fucoidan (200 μg/mL) in the upper chamber inserts coated with 50 μL of 1% Matrigel^®^ (BD Biosciences, San Jose, CA, USA). In the lower chamber, 600 μL medium containing 10% fetal bovine serum was used as a chemoattractant to encourage cell migration. After 24 h incubation, the cells on the upper surface of the inserts were gently removed with a cotton swab. All of the cells were stained using 0.1% crystal violet and counted in 5 fields under an inverted microscope. The independent experiments were repeated three times.

### 4.6. RNA Isolation and Reverse Transcription-Polymerase Chain Reaction (RT-PCR)

After treatment with fucoidan for 48 h, total RNA isolation was performed using Trizol (Invitrogen, Grand Island, NY, USA) according to the manufacturer’s instructions. Reverse transcription was carried out by using RevertAid™ Reverse Transcriptase (Fermentas, Amherst, NY, USA) and random primer mix (New England BioLabs, Schwalbach, UK). One microgram of total RNA from every sample was used for cDNA synthesis by Transcriptor First Strand cDNA Synthesis Kit (Roche, Foster City, CA, USA). Then, the cDNA was amplified by PCR with primers specific for DNMT3B, MTSS1, E-cadherin, N-Cadherin and glyceraldehyde-3-phosphate dehydrogenase gene (GAPDH). The product of GAPDH was used as loading control. The primers used were listed in Table 2.

**Table 2:** The primer sequences used in this study.

**DNMT3B**	Forward: AGGGAAGACTCGATCCTCGTC
	Reverse: GTGTGTAGCTTAGCAGACTGG
**MTSS1**	Forward: CAGTCCCAGCTTCGGACAAC
	Reverse: TGAGAGCAGATCCAATCTCCC
**E-cadherin**	Forward: CGAGAGCTACACGTTCACGG
	Reverse: GGGTGTCGAGGGAAAAATAGG
**N-cadherin**	Forward: TCAGGCGTCTGTAGAGGCTT
	Reverse: ATGCACATCCTTCGATAAGACTG
**GAPDH**	Forward: GGAGCGAGATCCCTCCAAAAT
	Reverse: GGCTGTTGTCATACTTCTCATGG
**miR-29b**	Forward: TGGTTTCATATGGTGGTTTA
	Reverse: ATAACCGATTTCAGATGGTG
**U6**	Forward: GTGCTCGCTTCGGCAGCACATATAC
	Reverse: AAAAATATGGAACGCTTCACGAATTTG

### 4.7. MicroRNA Profiling

Total RNA was isolated from HepG2 cells treated with vehicle or fucoidan at dose of 200 μg/mL for 48 h. The samples were then analyzed by using Affymetrix GeneChip miRNA 2.0 array (Affymetrix, Santa Clara, CA, USA) containing 4560 probe sets for human small RNAs. All steps of the procedure were performed according to the Affymetrix standardized protocol for miRNA 2.0 array.

### 4.8. Quantitative RT-PCR

For analyzing the expression of mature miR-29b, cDNA was synthesized using sequence-specific primers and the TaqMan MicroRNA Reverse Transcription kit (Applied Biosystems, Foster City, CA, USA). Each cDNA was amplified by qPCR using sequence-specific primers from the TaqMan MicroRNA Assays kit. qPCR was performed on an ABI 7500 real-time system (Applied Biosystems, Foster City, CA, USA). After normalization by the internal controls, the values for the vehicle-treated cells served as a basal level of miRNAs expression; ΔCt values (Ct_fucoidan_ − Ct_control_) were used to determine their relative expression as fold changes. U6 was used as the internal control for evaluation of miR-29b expression.

### 4.9. MicroRNA Mimic and Construct of DNMT3B 3′-UTR Vectors

MiR-29b mimic (5′-UAGCACCAUUUGAAAUCAGUGUU-3′) and scrambled control (5′-UUCUCCGAACGUGUCACGUTT-3′) were obtained from Dharmacon (Lafayette, CO, USA). The mimic is a single-stranded RNA oligonucleotide, which could bind to the complimentary, mature microRNA strand. The DNMT3B 3′-UTR of around 300 bps, which contained miR-29b binding site, was cloned into the downstream region of the luciferase gene to create pGL3-DNMT3B 3′-UTR. We used site-directed mutagenesis to generate pGL3-DNMT3B 3′-UTR-mut, in which miR-29b binding site were replaced with mutant site. For luciferase reporter experiments, a DNMT3B 3′-UTR segment of 978 bps was amplified by PCR from human genomic DNA and inserted into the pGL3-control vector with SV40 promoter (Promega, Madison, WI, USA) by using the XbaI site immediately downstream from the stop codon of luciferase. The following sets of primers were used to generate specific fragments: DNMT3B-3′-UTR Forward: 5′-GCTCTAGATAGGTAGCAACGTGGCTTTT-3′; DNMT3B-3′-UTR Reverse: 5′-GCTCTAGAGCCCCACAAAACTTGTCAAC-3′. Underlined sequences indicate the endonuclease restriction site.

### 4.10. Transfection and Luciferase Reporter Assay

The miR-29b or scrambled miRNA mimic (100 nM), DNMT3B 3′-UTR firefly luciferase report vectors (wild type or mutated type) and the control vector containing Renilla luciferase pRL-TK vector (Promega, Madison, WI, USA) were cotransfected into HCC cells using Lipofectamine-2000 transfection reagent (Invitrogen, Grand Island, NY, USA) according to the manufacturer’s protocol. The medium was changed after 16 h. The cells were cultured for 48 h and harvested for luciferase reporter assay. Firefly and Renilla luciferase activities were measured consecutively by using Dual-Luciferase^®^ Reporter Assay System (Promega, Madison, WI, USA). The experiments were performed in triplicate.

### 4.11. Western Blot Analysis

After treatment with fucoidan for 48 h, the cell lysates were separated by electrophoresis on sodium dodecyl sulfate-PAGE gel. Following gel transference to PVDF (Millipore, MA, USA), the antibodies against DNMT3B (ab16049, Abcam, Cambridge, MA, USA), MTSS1 (sc-101204, Santa Cruz, CA, USA), E-cadherin (sc-8426, Santa Cruz, CA, USA), N-Cadherin (sc-8424, Santa Cruz, CA, USA), TGF-βR1 (sc-398, Santa Cruz, CA, USA), TGF-βR2 (sc-220, Santa Cruz, CA, USA), Phospho-Smad2/3 (No. 8828, Cell Signaling Technology, Danvers, MA, USA), Smad4 (No. 9515, Cell Signaling Technology, Danvers, MA, USA), Smad7 (ab55493, Abcam, Cambridge, MA, USA), MMP2 (sc-10736, Santa Cruz, CA, USA), MMP9 (sc-6840, Santa Cruz, CA, USA), TIMP-1 (sc-5538, Santa Cruz, CA, USA) and GAPDH (sc-25778, Santa Cruz, CA, USA) were used. Subsequently, suitable secondary antibodies were applied and bands for specific molecules were detected by enhanced chemiluminescence (ECL; Millipore, Billerica, MA, USA).

### 4.12. Statistical Analysis

All of the data are expressed as the mean ± standard deviation (SD). Significant differences between two groups were determined by *t* test analyses using Microsoft Excel. A *p*-Value of less than 0.05 was considered statistically significant (* *p* < 0.05, ** *p* < 0.01, compared with the control group).

## 5. Conclusions

The results obtained in the present study demonstrate that fucoidan increased miR-29b to suppress the DNMT3B, which resulted in the upregulation of MTSS1. On the other hand, fucoidan also down-regulated the TGF-β signaling pathway of HCC cells. These effects led to inhibition of EMT and prevention of extracellular matrix degradation, by which the invasion activity of HCC cells was diminished. As fucoidan has advantages of low toxicity, oral bioavailability, and multiple mechanisms of action [29], future development as an integrative therapeutics for improving the treatment outcome of patients with HCC is warranted.
